# Designing Tunable GelMA Hydrogels by Integrating Mammalian and Non-Mammalian Gelatins

**DOI:** 10.3390/gels12060540

**Published:** 2026-06-15

**Authors:** Cristina Padilla, Vanessa Campos, Eduardo González, Francisco Kirhman, Javier Enrione

**Affiliations:** 1 Biopolymer Research & Engineering Laboratory (BIOPREL), Escuela de Nutrición y Dietética, Facultad de Medicina, Universidad de Los Andes, Santiago 7620086, Chile; cpadillar@uandes.cl; 2 Centro de Investigación e Innovación Biomédica (CIIB), Universidad de los Andes, Santiago 7620086, Chile; eduardogonzalezmienert@gmail.com (E.G.); francisco.osorio@ug.uchile.cl (F.K.); 3 Laboratorio de Ingeniería de Polímeros, Departamento de Ingeniería Química, Biotecnología y Materiales, Facultad de Ciencias Físicas y Matemáticas, Universidad de Chile, Santiago 8370448, Chile; vanessacampos@ug.uchile.cl

**Keywords:** GelMA, gelatin hydrogels, salmon gelatin, porcine gelatin, mechanical properties, degree of substitution, controlled release, tissue engineering

## Abstract

Modulating the physical crosslink architecture of gelatin methacryloyl (GelMA) hydrogels without altering total polymer concentration or introducing exogenous components remains a central challenge in biomaterial design. Here, we present a source blending strategy in which porcine skin gelatin (PG) and salmon skin gelatin (SG), two gelatins with markedly different proline and hydroxyproline contents, are combined at seven compositional ratios (PG weight fractions 0–1.0) and subsequently functionalized to GelMA under standardized conditions (8% *v*/*v* methacrylic anhydride, 60 °C, 3 h). Near-complete degrees of substitution (95–98%) were achieved across all formulations, as confirmed by both TNBS and ^1^H-NMR analyses. In the parent gelatin mixtures, increasing PG fraction progressively increased viscosity, elastic modulus (G′), gelation temperature (Tgel), and compression modulus at 4 °C, with DSC revealing independent SG (0–15 °C) and PG (20–40 °C) endothermic transitions that suggest partial hindrance of PG triple-helix formation by high SG fractions. These composition-dependent trends were preserved after functionalization to GelMA, albeit with attenuated physical crosslinking due to steric impairment by the methacrylate groups. Photocrosslinked GelMA hydrogels fabricated after pre-incubation at 4 °C exhibited systematically higher compression moduli and lower swelling degrees with increasing PG content, demonstrating that the PG/SG ratio provides an effective means for independently tuning hydrogel mechanics and mesh architecture. In vitro release assays using Rhodamine 6G further demonstrated that pre-incubation at 4 °C prior to photocrosslinking effectively modulates transport kinetics in SG-PG GelMA hydrogels. This strategy delayed characteristic release times and constrained Weibull shape parameters to the anomalous-transport regime (0.75 < β < 1), where diffusion is governed by network chain relaxation. This effect was most pronounced in the 0.4SG:0.6PG formulation, where lower SG content permitted unhindered triple-helix formation, as corroborated by DSC and compression studies. Ultimately, adjusting the pre-incubation temperature and gelatin source combination provides a straightforward, processing-additive-free strategy to achieve programmable release profiles via controlled matrix tortuosity.

## 1. Introduction

Gelatin is a versatile hydrocolloid derived from the partial hydrolysis and thermal denaturation of collagen [1], obtainable from bovine, porcine, fish, and poultry sources, each yielding materials with distinct physicochemical properties depending on the animal source and extraction conditions [2]. Two principal types are recognized: type A, produced by acid hydrolysis (isoelectric point pH 6–9, predominantly from porcine skin [3]), and type B, produced by alkaline hydrolysis (isoelectric point pH 5–6 [4,5]). At the molecular scale, gelatin comprises α-, β-, and γ-chains [4,6], where α-chains adopt a polyproline II conformation [7,8], sustained by the repetitive Gly-X-Y tripeptide sequence, with proline (Pro) and hydroxyproline (Hyp) most frequently occupying the X and Y positions [9]. This sequence governs the thermoreversible formation of collagen-like triple-helix structures upon cooling, whose extent and thermal stability are directly proportional to total Pro + Hyp content [10,11]. Cold-water fish gelatins, including those derived from salmon skin, contain markedly lower Pro + Hyp levels than mammalian gelatins, resulting in lower Bloom values, reduced gel strength, and melting points that fall well below physiological temperature [11,12,13]. These properties, however, confer distinct processing advantages: salmon skin gelatin remains in the sol state at 5 °C [14,15,16], making it particularly attractive for biofabrication approaches that require low-viscosity inks at near-ambient temperatures. Moreover, its use offers eco-friendly valorization of a major aquaculture by-product and avoids the religious and ethical restrictions associated with mammalian-derived gelatins [10,17,18,19].

Interest in gelatin for biomedical applications has grown considerably, driven by its excellent biocompatibility, biodegradability, and intrinsic cell-adhesion motifs that recapitulate key features of the native extracellular matrix (ECM) [20,21,22]. Nevertheless, its low melting temperature and susceptibility to enzymatic degradation under physiological conditions limit direct use in tissue engineering scaffolds [23,24]. Chemical modification into gelatin methacryloyl (GelMA) overcomes these limitations by introducing photocrosslinkable methacrylate groups that enable the formation of covalently stabilized hydrogel networks with independently tunable stiffness, porosity, and degradation rate [25,26,27]. The resulting hydrogels retain the ECM-mimetic properties of native gelatin, supporting cell adhesion, proliferation, and spreading, and are compatible with a wide range of fabrication strategies, including bioprinting and microfabrication [28]. Mechanical and structural properties can be further modulated by adjusting the degree of substitution (DS), polymer concentration, and photocrosslinking conditions [29,30,31], though high GelMA concentrations increase network stiffness at the expense of porosity and cell infiltration [32]. Strategies to decouple these trade-offs include the incorporation of nanomaterials [25,32], optimization of the functionalization protocol [33], and the induction of physical crosslinks through temperature-driven triple-helix formation prior to photopolymerization [34,35]. This last strategy is particularly relevant when working with gelatin mixtures, as the source-dependent differences in Pro + Hyp content create compositionally programmable physical crosslink densities. Geonzon et al. [36] demonstrated that blending porcine skin gelatin with cold-water fish gelatin reinforces the gelation temperature and complex modulus even at low porcine fractions, with the extent of reinforcement and the resulting network architecture, ranging from intertwined hybrid triple-helices to isolated, separate polymer networks, governed by the cooling rate applied during gelation [36]. This thermally programmable interspecies co-aggregation mechanism offers a compelling strategy for modulating the physical crosslink network in GelMA hydrogels without altering total polymer concentration or introducing exogenous materials.

Despite the promise of GelMA, its production still lacks standardization: reported synthesis protocols vary widely in reaction pH, temperature, and duration [37,38,39,40,41,42], and these variables have a significant impact on the degree of substitution, photopolymerization efficiency, and the resulting mechanical performance [43]. Thorough characterization of GelMA after synthesis is therefore essential before any inference about hydrogel behavior can be drawn.

From a sourcing perspective, while the previous literature has evaluated the use of cold-water fish gelatin for GelMA production, a major limitation in these existing studies is the near-complete lack of structural and species traceability. Available reports have relied on generic commercial fish gelatin without information regarding its specific source, molecular weight distribution, or amino acid composition [19,39]. This absence of structural characterization introduces significant experimental variability, as these characteristics dictate the final hydrogels’ mechanical integrity and thermal properties. To overcome these limitations, we intentionally introduce a traceable alternative by utilizing gelatin directly derived from salmon skin. Salmon skin represents an abundant and underutilized co-product of the aquaculture industry, offering collagen content among the highest reported for fish-derived by-products [44]. The highly controlled feeding and husbandry protocols of intensive salmon farming further reduce the compositional variability that typically complicates batch-to-batch reproducibility in gelatin extraction, making salmon an attractive and sustainable raw material for biomedical-grade GelMA.

Building on this context, the present work comprehensively investigates the gelation behavior and physicochemical properties of hydrogels derived from mixtures of well-characterized porcine and salmon gelatins across a range of compositional ratios, and their corresponding GelMA derivatives. We demonstrate how the PG-SG ratio can be exploited as a tunable structural parameter, independent of total polymer concentration or chemical crosslink density, to produce GelMA hydrogels with systematically varied triple-helix content, mechanical stiffness, swelling behavior, and controlled-release kinetics. The approach offers a simple, cost-effective route to structurally modulated hydrogels based on naturally derived materials.

## 2. Results and Discussion

### 2.1. Gelatin Characterization

Both porcine and salmon gelatins exhibited high purity, with protein contents exceeding 90% in both cases (Table S1). Salmon gelatin (SG) showed slightly lower protein and higher ash content compared to commercial porcine gelatin (PG) (Table S1). Consistent with previous reports [45], SG contained significantly lower concentrations of proline (Pro) and hydroxyproline (Hyp) compared to PG (Table S2), reflecting the well-established compositional differences between cold-water fish and mammalian gelatins.

### 2.2. Gelatin Mixtures Characterization

**Figure 1:**
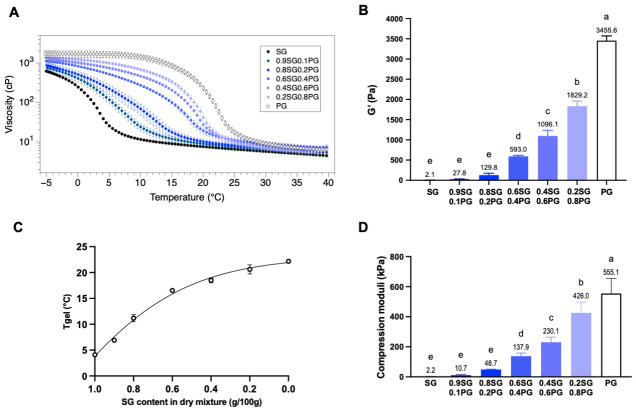
Rheological and mechanical characterization of gelatin suspensions at 7% *w*/*v* pH 7.4 from SG-PG mixtures. (**A**) Representative viscosity cooling ramps (n = 3). (**B**) Elastic modulus (G′) at 4 °C obtained from oscillation cooling ramp measurements (n = 3). (**C**) Gelation temperature (Tgel), as determined as the G′ and G″ crossover (n = 3). (**D**) Compression modulus after gelation at 4 °C (n = 6). Different letters indicate statistically significant differences between groups (*p* < 0.05). Groups sharing at least one common letter are not significantly different (*p* ≥ 0.05), as determined by one-way ANOVA followed by Tukey’s multiple comparisons test.

Gelatin mixture samples were prepared and characterized at 7% *w*/*v*, a concentration chosen for its comparability to standard Bloom value determinations [17]. Increasing PG proportions in gelatin mixtures increased viscosity across all evaluated temperature ranges (Figure 1A), accompanied by increases in elastic modulus (G′), compression modulus at 4 °C (Figure 1B,D and Figure S1), and in the gelation temperature (Tgel) (Figure 1C and Figure S1). This suggests that PG effectively reinforces the gelatin mixture’s molecular structure even at low contents, as evidenced by G′, Tgel, and compression modulus of the 0.9SG0.1PG sample. Interestingly, this reinforcement is not linear (Figure 1C), suggesting that molecular interactions or entanglements may be occurring.

**Figure 2:**
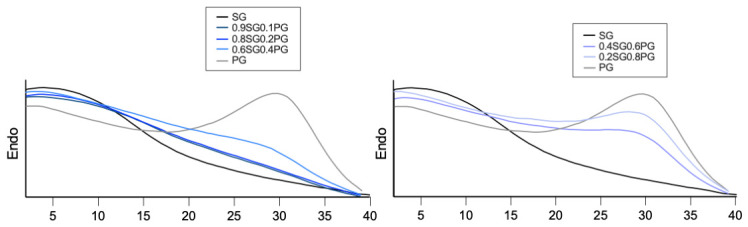
Representative endotherms of gelatin suspensions at 7% *w*/*v* pH 7.4 from SG-PG mixtures.

Thermal properties determined by DSC revealed no unique transition temperature at the tested cooling rate; instead, separate transitions from SG (0–15 °C) and PG (20–40 °C) were identified (Figure 2 and Table 1), suggesting limited inter-species chain interaction, with high SG fractions likely hindering PG triple-helix formation. Since our DSC data show separate endothermic transitions for PG and SG under the tested conditions, we cannot confirm the presence of interspecies chain aggregation. This contrasts with the work of Geonzon et al. [36], who showed a single endothermic transition dependent on the relative fractions of fish scale and porcine gelatins using micro-DSC. However, in that study, both gelatins had similar molecular weights, whereas our SG has a lower molecular weight than PG [45], potentially influencing the extent of SG-PG interspecies chain interactions. Overall, under the cooling conditions employed in this study (3 °C/min), hydrogels containing both mixed and distinct PG and SG helical structures can be obtained.

### 2.3. GelMA Suspensions Characterization

After functionalization, DS was determined by TNBS and ^1^H-NMR, with both methods yielding very similar results and near-complete functionalization (95–98%) across all samples (Table 2). By combining these two independent methodologies, we ensured a highly rigorous quantification of the DS. Crucially, both characterization techniques specifically quantify the modification of gelatin’s primary amine groups (lysine residues). While other hydroxyl or thiol-containing amino acids (hydroxylysine, tyrosine, serine, threonine, and cysteine) can undergo minor functionalization, this side-reaction typically accounts for less than 10% of the overall modification under these conditions. Furthermore, this near-complete amine substitution does not compromise the biological functionality of the hydrogel, as the key cell-adhesive motifs naturally present in gelatin, including RGD (Arg-Gly-Asp) sequences, do not contain reactive primary amines and therefore remain unaffected by the methacrylation process. Consistent with previous reports, highly functionalized GelMA networks do not exhibit cytotoxicity. Instead, a high degree of substitution (DS) increases crosslinking density and matrix stiffness, which may influence cell migration and pore size-dependent matrix remodeling while still supporting robust cell attachment and high cell viability.

**Figure 3:**
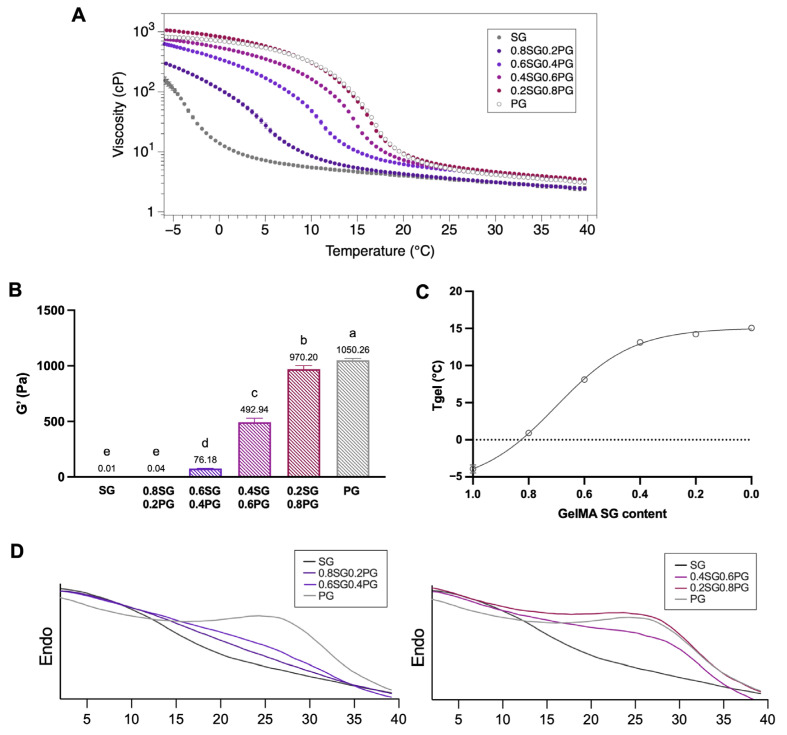
Rheological and thermal characterization of GelMA suspensions at 7% *w*/*v* pH 7.4 obtained from SG-PG mixtures. (**A**) Representative viscosity cooling ramps (n = 3). (**B**) Elastic modulus (G′) at 4 °C obtained from oscillation cooling ramp measurements (n = 3–4 per group). (**C**) Gelation temperature (Tgel), as determined by the G′ and G″ crossover (n = 3–4 per group). Different letters indicate statistically significant differences between groups (*p* < 0.05). Groups sharing at least one common letter are not significantly different (*p* ≥ 0.05), as determined by one-way ANOVA followed by Tukey’s multiple comparisons test. (**D**) Representative DSC endotherms of the samples.

**Figure 4:**
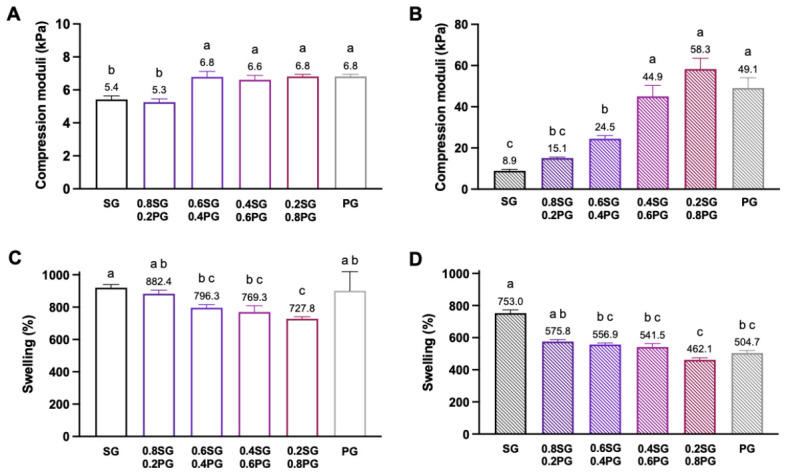
Mechanical and structural characterization of GelMA hydrogels (7% *w*/*v* pH 7.4) obtained from SG-PG mixtures (**A**) Compression modulus of hydrogels photocrosslinked at 37 °C. (**B**) Compression modulus of hydrogels photocrosslinked after 1 h pre-incubation at 4 °C (n = 3–4 per group). (**C**) Swelling of hydrogels photocrosslinked at 37 °C. (**D**) Swelling of hydrogels photocrosslinked after 1 h pre-incubation at 4 °C (n = 3 per group). Each measurement was performed in triplicate. Different letters indicate statistically significant differences between groups (*p* < 0.05). Groups sharing at least one common letter are not significantly different (*p* ≥ 0.05), as determined by one-way ANOVA followed by Tukey’s multiple comparisons test or Kruskal–Wallis test with Dunn’s multiple comparisons (for swelling assays).

Rheological characterization showed similar trends to gelatin mixtures: increasing PG weight fraction increased viscosity, G′ at 4 °C, and Tgel of GelMA samples (Figure 3A–C and Figure S2). However, functionalization decreased overall viscosity, G′ at 4 °C, and Tgel compared to original gelatin mixtures (Figure 1 and Figure 3), attributed to steric impairment due to the presence of methacryloyl groups and decreased molecular weight during the functionalization reaction. DSC revealed similar trends, displaying an overall lower triple-helix content due to chain destructuring (Table 3 and Figure 4A). For example, in the case of the 0.8SG0.2PG GelMA sample, the transition related to porcine gelatin cannot be detected. Further evidence of this chain disruption can be identified in the 0.2SG0.8PG GelMA sample, where no significant differences in rheological behavior or triple-helix content were observed compared to porcine GelMA (Table 3 and Figure 3D), suggesting that the maximum triple-helix content was reached in the evaluated system.

Aiming to promote biomimicry as well as to discern the structural and rheological differences between PG, SG, and SG-PG blends, we pre-incubated our samples at 4 °C for 1 h prior to photocrosslinking. This thermal pre-conditioning produced hydrogels with a significantly higher compression modulus than their 37 °C counterparts, with stiffness increasing progressively alongside higher PG content (Figure 4A,B). Although no differences were found in the thermal characterization between 0.2SG0.8PG GelMA and porcine GelMA, a higher compression modulus was observed in the former, potentially due to the contribution of salmon GelMA triple helices at 4 °C. The presence of PG also reduced the swelling degree, an effect that was more pronounced at higher PG contents and significantly lower than that observed in hydrogels prepared at 37 °C (Figure 4C,D). This behavior suggests a lack of an ordered physical network assembly when crosslinking is performed at 37 °C, contrasting with the structured networks formed at 4 °C. Overall, under the cooling conditions used (3 °C/min) in this study, GelMA hydrogels with differential triple-helix content, mechanical properties, and swelling degree can be obtained by modulating the SG-PG ratio: higher PG content yields hydrogels with higher compression modulus and lower swelling. Notably, utilizing this thermal pre-conditioning step to drive physical triple-helix assembly allows for the safe enhancement and tuning of hydrogel mechanics while minimizing synthetic chemical modifications. This avoids potential toxicity risks associated with increasing the DS, increasing chemical photoinitiator concentrations, or increasing UV light exposure. Consequently, the structural variations observed due to differential SG-PG proportions and physical crosslinking induction suggest a strong potential for actively modulating drug release profiles, as investigated in Section 2.4.

### 2.4. Rhodamine 6G Loading and Release

**Figure 5:**
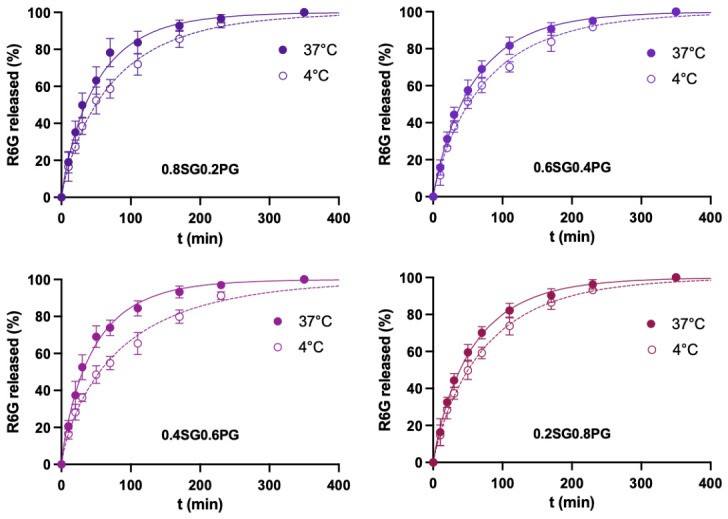
Release profiles of Rhodamine 6G (R6G) from GelMA hydrogels submerged in PBS 1×. Hydrogels were produced by pre-incubation at 37 °C (closed circles) or 4 °C (open circles) prior to photocrosslinking. Values represent the mean of 6 hydrogels per sample.

Rhodamine 6G (R6G) was used as a model molecule to evaluate the loading and release capacity of the GelMA hydrogels, as it has been previously employed in analogous systems [46]. Similar cumulative release profiles were obtained for all GelMA formulations (Figure 5). The pre-incubation temperature prior to photocrosslinking markedly modulated R6G delivery: hydrogels pre-incubated at 4 °C exhibited slower cumulative R6G release compared to those pre-incubated at 37 °C (Figure 5).

The Weibull model was selected to describe the release kinetics of the different GelMA formulations, yielding an excellent fit across all datasets (R^2^ ≥ 0.995) (Table 4). The model parameters considered most critical for our samples are α and β. The parameter α represents the time required to reach a cumulative molecule release fraction of 63.2%, a value derived from the mathematical structure of the exponential Weibull distribution. Meanwhile, β defines the physical transport mechanism of the model molecule and dictates the geometric shape of the release curve [47,48]. This model showed an increase in *α* values when physical crosslinking was promoted, indicating overall slower release kinetics and greater resistance to the diffusion process, likely arising from physical or chemical barriers such as matrix tortuosity or polymer-molecule interactions [47,48].

The values obtained for β had a range of 0.75 < β < 1, suggesting that the transport mechanism within these hydrogels is not purely dominated by Fickian diffusion, indicating anomalous transport that combines Fickian diffusion with structural relaxation of the polymer network [49,50]. This behavior reflects the dual network architecture of GelMA hydrogels, in which covalent crosslinks established during photopolymerization coexist with physically associated triple-helix domains derived from the gelatin chains.

These results collectively demonstrate that the pre-incubation temperature, and thus the extent of physical crosslinking prior to photopolymerization, is a simple and effective lever for tuning the drug release profile of SG-PG GelMA hydrogels. While the overall differences among different SG-PG proportions remained relatively subtle, the 0.4SG0.6PG formulation exhibited a greater increase in β at 4 °C, with a more pronounced disparity between its 37 °C and 4 °C kinetic profiles. Given that higher SG contents appeared to hinder the assembly of PG triple-helical structures, as corroborated by our DSC and compression tests, the 0.4SG0.6PG formulation stands out as the most structurally responsive candidate and a compelling starting point for further optimization since it is uniquely characterized by its high sensitivity to thermal pre-conditioning. This behavior suggests that this formulation undergoes substantial, tunable structural rearrangements within its macromolecular network, a mass transport mechanism governed by a greater contribution from matrix relaxation and network-related effects compared to the other formulations. Accordingly, this formulation offers a highly versatile platform where release kinetics can be actively modulated through processing conditions, rather than being restricted to pure passive diffusion.

Regarding our molecule release model, we are aware of the inherent structural and electrostatic limitations of using R6G. Consequently, future validation using clinically relevant therapeutic molecules, such as specific small-molecule drugs or macromolecular biologics, is required to comprehensively establish the translation of these hybrid matrices into targeted drug delivery applications. Furthermore, because the thermal pre-conditioning step physically reinforces the hydrogel network, future work will explore the feasibility of reducing the UV exposure time and/or utilizing GelMA with a lower DS. Optimizing these parameters could minimize chemical and radical-induced cellular stress even further, maximizing the cytocompatibility of these hybrid matrices for advanced tissue engineering and the development of drug delivery systems.

## 3. Conclusions

This study demonstrates that blending porcine skin gelatin (PG) and salmon skin gelatin (SG) at controlled compositional ratios constitutes a straightforward, additive-free strategy for engineering GelMA hydrogels with tunable physical crosslinking density, mechanical stiffness, swelling behavior, and compound release kinetics, without modifying total polymer concentration or the degree of chemical functionalization. Gelatin source composition determines the physical crosslink landscape. SG, with its lower Pro + Hyp content, remains largely in the sol state at biofabrication-relevant temperatures and contributes minimal physical crosslinking, while PG forms thermally stable triple helices that reinforce the network in a dose-dependent, non-linear fashion. DSC confirmed that under the tested cooling conditions, PG and SG chains form largely independent helical populations, with high SG fractions partially hindering PG triple-helix nucleation, a behavior distinct from co-aggregation systems where both gelatins share similar molecular weights. The functionalization of gelatin mixtures attenuated their ability to form physical crosslinks, reducing viscosity, G′, and Tgel in all samples relative to parent gelatins, consistent with steric impairment of triple-helix formation. However, compositional trends were preserved after functionalization, validating the source ratio as a reliable pre-functionalization control parameter. The pre-incubation temperature prior to photocrosslinking acts as a direct lever for modulating molecule delivery in SG-PG GelMA hydrogels. Promoting physical crosslinking via low-temperature pre-incubation prolonged the characteristic release time, probably by introducing structural barriers like matrix tortuosity. Modeled release profiles confirmed anomalous transport governed by network chain relaxation, consistent with the dual network architecture of the hydrogels, in which covalent crosslinks coexist with physically associated triple-helix domains. Future work should address cell compatibility across the formulation space, explore slower cooling rates to evaluate the promotion of interspecies triple-helix formation, and validate release behavior with biologically relevant small molecules. Additionally, the physical reinforcement provided by thermal pre-conditioning opens opportunities to investigate the use of GelMA materials with lower DS and minimized UV exposure times, thereby further reducing potential chemical and radical-induced cytotoxicity.

## 4. Materials and Methods

### 4.1. Materials

Atlantic salmon (*Salmo salar*) skins were provided by AquaChile, Cardonal Plant, Puerto Montt, Chile. Sodium hydroxide (≥99.0%), glacial acetic acid, phosphate-buffered saline (PBS) 10×, chlorohydric acid (HCl), sodium dodecyl sulfate (SDS), and deuterium oxide (≥99.9% deuteration) were purchased from Merck (Darmstadt, Germany). Gelatin from porcine skin (gel strength ~300 g Bloom, Type A), methacrylic anhydride (≥94%), 2-hydroxy-4′-(2-hydroxyethoxy)-2-methylpropiophenone (IRGACURE 2959), glycine, serine, 2,4,6-trinitrobenzenesulphonic acid (TNBS), sodium bicarbonate, and rhodamine 6G were purchased from Sigma Aldrich (St. Louis, MO, USA).

### 4.2. Salmon Gelatin Extraction

Salmon gelatin was extracted following Zhou & Regenstein [51] with modifications as described in Padilla et al. [45]. Briefly, salmon skins were cleaned and cut into ~3 cm^2^ pieces, subjected to two pretreatments in 0.1 M NaOH at 10 °C for 1 h each, and then 0.05 M acetic acid under similar conditions, with tap water washes between steps. Gelatin was extracted at pH 4.0 (acetic acid) at 60 °C for 4 h, vacuum filtered and dried at 60 °C for 72 h. The resulting gelatin was ground and stored until use.

### 4.3. Gelatin Composition

Composition was determined by proximate analysis and amino acid profiling according to AOAC methods [52]. Moisture content was measured gravimetrically after drying the samples at 105 °C for 24 h. Crude fat content was determined using Soxhlet extraction, and total protein content was evaluated via the Kjeldahl method, utilizing a nitrogen-to-protein conversion factor of %N × 5.55. Ash content was measured by calcination in a muffle furnace at 550 °C, while the non-nitrogenous fraction was estimated by difference [17]. For amino acid profiling, key residues (Gly, Pro, and Hyp) were quantified using high-performance liquid chromatography (HPLC) [53]. Samples were subjected to acid hydrolysis using 6 N HCl at 110 °C for 24 h, followed by pre-column derivatization with phenylisothiocyanate. Separation was performed on a Luna RP18 column (Phenomenex, Torrance, CA, USA) utilizing an HPLC system equipped with a Waters 600 controller and a Waters 996 photodiode array detector (DAD) (Waters, Milford, MA, USA) set at 254 nm. All proximate and amino acid values were expressed as g/100 g of sample.

### 4.4. Production of Gelatin Mixtures

Dry salmon (SG) and porcine (PG) gelatins were mixed in different weight proportions. PG weight fractions: 0, 0.1, 0.2, 0.4, 0.6, 0.8, and 1.0, yielding seven samples.

### 4.5. Gelatin Suspensions Functionalization

SG-PG mixtures were suspended at 10% *w*/*v* in PBS 1× (pH 7.4) and functionalized with methacrylic anhydride (MA) at 8% *v*/*v*, pH ~4, 60 °C for 3 h. The reaction was stopped by dilution with three times the initial PBS 1× volume. Suspensions were vacuum filtered (22 µm, Whatman, Maidstone, UK) and diafiltered in a Tangential Flow Filtration (TFF) system (SartoFlow^®^ Smart, Sartorius, Göttingen, Germany) equipped with a 10 kDa cutoff Sartocon Hydrosart^®^ (Göttingen, Germany) stabilized cellulose-based membrane cassette. To maintain GelMA in the sol state without exceeding the membrane’s maximum temperature operating limit (50 °C), distilled water at 60 °C was fed into the system, maintaining the effective operating retentate temperature between 30 °C and 45 °C. The process was conducted at a 20% crossflow rate and an operating pressure of 1.5 bar until the filtrate conductivity stabilized at ~100 μS. Finally, the purified GelMA suspensions were collected, freeze-dried, and stored at 4 °C for future use.

### 4.6. Degree of Substitution by TNBS

The TNBS assay was performed as described by Claaßen et al. [54], with minor modifications. Samples were dissolved at a concentration of 20 mg/mL in a sodium bicarbonate solution (4% *w*/*v*, pH 6.0). Aliquots of 25 µL were subsequently mixed with 25 µL of the sodium bicarbonate buffer and 25 µL of a 0.1% (*v*/*v*) TNBS solution. The reaction mixture was incubated at 37 °C for 2.5 h and protected from the light. After incubation, the reaction was stopped by adding 25 µL of a 10% (*w*/*v*) SDS solution and 25 µL of 1 M HCl. The absorbance of the resulting solution was measured at 335 nm using a microplate reader (Spark, Tecan, Männedorf, Switzerland). A glycine standard curve (12.5–100 µg/mL) was used. Measurements were performed in triplicate. The DS of amine groups in the GelMA samples was determined with respect to an average value of amine groups of the corresponding unmodified gelatin as follows:

$$DS\%=\;\left[1-\left(\frac{GelF}{Gel}\right)\right]\times 100$$

where *GelF* = functionalized gelatin, and *Gel* = unmodified gelatin.

### 4.7. Degree of Substitution by Proton Nuclear Magnetic Resonance (^1^H-NMR) Spectroscopy

Samples at 6 mg/mL in D_2_O were analyzed on a Bruker Avance II+ 500 MHz spectrometer (B500, Bruker, Karlsruhe, Germany). All measurements were performed at 25 °C and 16 scans were acquired. Data were processed with MestReNova14 (Mestrelab, A Coruña, Spain). Spectra were normalized to the phenylalanine signal (6.9–7.5 ppm). The lysine methylene peak (2.95–3.05 ppm) was integrated, using manual integration of peaks of interest with automatic linear correction. Measurements were performed once to confirm TNBS results. The DS was calculated as described by Hoch et al. [55]:

$$DS\;\left(\%\right)=\left(1-\frac{lysine\;methylene\;peak\;area\;of\;GelMA}{lysine\;methylene\;peak\;area\;of\;gelatin}\right)\times 100$$

### 4.8. Rheological Characterization

Gelatin mixtures and GelMA samples prepared at 7% *w*/*v* in PBS 1× pH 7.4 were characterized using a Discovery HR-2 rheometer (TA Instruments, New Castle, DE, USA). After the determination of the linear viscoelastic region (LVR), oscillatory measurements during cooling (40 to −5 °C, 3 °C/min) at 1% deformation and 1 Hz were performed. A 50 mm parallel plate geometry with a 300 µm gap was used, as well as a solvent trap to prevent water evaporation during the analysis. Gelation temperature (Tgel) was determined as the elastic modulus (G′) and loss modulus (G″) crossover. Viscosity was measured at 800 s^−1^ using a 40 mm, 0.5° cone geometry. Measurements were performed at least in triplicate. The viscosity of the suspensions was determined using the same temperature range and rate as oscillatory measurements, with a shear rate of 800 s^−1^. A 40 mm diameter and 0.5° angle cone geometry with a 50 µm gap were used.

### 4.9. Mechanical Characterization of Gelatin Mixtures

Gelatin mixtures at 7% *w*/*v* PBS 1× pH 7.4 were loaded on a 40 mm parallel plate (2 mm gap) preheated to 37 °C using a Discovery HR-30 rheometer (TA Instruments, New Castle, DE, USA), conditioned at 4 °C for 480 s, and then subjected to unconfined linear compression at 10 µm/s at 4 °C. Compression modulus was calculated from the initial linear region up to 4% strain.

### 4.10. Thermal Properties by Differential Scanning Calorimetry (DSC)

Thermal transitions were determined with a DSC-1 (Mettler Toledo, Greifensee, Switzerland) calibrated with indium (T_m_ = 156.6 °C, ΔH = 28.4 J/g). Approximately 90 µL of a 10% *w*/*v* pH 7.4 suspension was loaded into 100 µL aluminum pans. The thermal program was as follows: heating to 40 °C at 10 °C/min (hold 3 min); cooling to −15 °C at 3 °C/min (hold 3 min); heating to 40 °C at 3 °C/min. ΔH_m_ was estimated from the main endotherm.

### 4.11. GelMA Hydrogel Fabrication

GelMA suspensions were prepared at 7% (*w*/*v*) in 1× PBS supplemented with 0.3% (*w*/*v*) Irgacure 2959 and adjusted to pH 7.4. The photoinitiator was previously dissolved in PBS 1× by heating to 80 °C under continuous stirring until complete dissolution and solution clarity were achieved [56]. The final suspensions were maintained at 37 °C for at least one hour and immediately poured into PDMS molds (10 mm diameter, 2 mm height) for crosslinking or poured into PDMS molds, pre-incubated at 4 °C for 1 h (FOC 215E, VELP Scientifica, Usmate, Italy) and then immediately crosslinked. Crosslinking was achieved by exposing the samples for 2 min to UV light (365 nm) using a high-power spot-curing system (12 W/cm^2^ Cool Cure 395, Lesco UV, American Ultraviolet, Paramount, CA, USA). Samples were positioned at a distance of 4 cm from the light probe, resulting in an effective surface irradiance of approximately 100–150 mW/cm^2^, in line with previous acellular characterization protocols [57,58].

### 4.12. Mechanical Characterization of GelMA Hydrogels

Hydrogels were tested using a TA.XT2 Plus texture analyzer (Stable Micro Systems, Surrey, UK) in an unconfined compression setting. Hydrogels were compressed 1 mm at a compression rate of 0.2 mm/s using a 1 cm diameter cylindrical probe at room temperature. The compression modulus was calculated from the initial slope of the stress/strain curve that included data up to 5% strain.

### 4.13. Swelling

The swelling degree was quantified by first freezing the hydrogels through a stepwise cooling protocol (4 °C for 2 h, −20 °C for 24 h, and −80 °C for 24 h) and subsequent freeze-drying for 24 h. Upon recording the dry weight, the samples were rehydrated in 1× PBS at 37 °C for 24 h. After reaching equilibrium, the hydrogels were removed from the medium, gently blotted to remove excess surface moisture, and re-weighed to obtain the swollen mass. The final swelling ratio was calculated as follows:

$$Swelling\;\%=\left(\frac{Weq-Wi}{Wi}\right)\times 100$$

where *Weq* and *Wi* are the weights of the swollen sample after equilibrium and the initial dried sample, respectively.

### 4.14. Controlled Release Assays

Rhodamine 6G (R6G) was used as a model molecule, as it has been previously employed in controlled release assays from hydrogels [46]. Hydrogels were prepared as in Section 4.11, cooled through a stepwise cooling protocol (4 °C for 2 h, −20 °C for 24 h, and −80 °C for 24 h), freeze-dried, and rehydrated in PBS 1× for 24 h at 37 °C. After rehydration, hydrogels were immersed in R6G loading solution (0.5 mg/mL in PBS 1×) for 72 h at 37 °C. The release at 37 °C was monitored in 2.5 mL PBS 1× by withdrawing 100 µL aliquots at 10, 20, 30, 50, 110, 170, 230, 350 min, replacing with an equal volume of fresh PBS 1× to maintain sink conditions. R6G was quantified spectrophotometrically at 525 nm (Infinite 200 Pro, Tecan, Männedorf, Switzerland). Cumulative released mass was calculated as follows:

$$M_{\mathrm{cum}}\left(n\right)=V_{t}C_{n}+\sum_{i=1}^{n-1}V_{s}C_{i}$$

where *V_t_* = 2.5 mL (total volume), and *V_s_* = 0.1 mL (aliquot volume).

### 4.15. Weibull Kinetic Modeling

To characterize the release kinetics of R6G from the GelMA hydrogels, the Weibull model [47] was fitted to the cumulative release data normalized to the release value at 350 min, which was assumed to represent the plateau (maximum) release since no significant increase was observed at longer times. The Weibull equation corresponds to the following:

$$M=M_{\infty }\left[1-e^{-(\frac{t-T}{\alpha }{)}^{\beta }}\right]$$

where *α* is the characteristic release time (time for 63.2% of drug release), *β* is the shape exponent that governs the release mechanism, and *T* is the lag time prior to detectable release [48,49], which was fixed at 0 due to the immediate release of rhodamine upon contact with PBS 1×. Model parameters were estimated by nonlinear regression, and the fit accuracy was assessed by the coefficient of determination (R^2^).

### 4.16. Statistical Analysis

The data were summarized in tables by calculating the mean and standard deviation. The number of replicates for each experiment is indicated in each figure legend. For inferential statistics analysis, the comparison of the means of each experimental group was established using one-way ANOVA with Tukey’s multiple comparisons or using Kruskal–Wallis test with Dunn’s multiple comparisons for non-parametric data. Statistical analyses were performed with a confidence level of 95%, using the software Graph Pad Prism V.10.

## Figures and Tables

**Table 1:** Melting enthalpy (∆H_m_) values, determined by the integration of the endotherms derived from salmon gelatin (SG endo) and porcine gelatin (PG endo) in SG-PG mixtures at 10% *w*/*v* pH 7.4 (n = 5–6 per group). Different letters indicate statistically significant differences between groups (*p* < 0.05). Groups sharing at least one common letter are not significantly different (*p* ≥ 0.05), as determined by one-way ANOVA followed by Tukey’s multiple comparisons test.

Gelatin Sample	ΔH_m_ (Jg^−1^)	
	SG Endo	PG Endo
SG	4.00 ± 0.14 ^a^	-
0.9SG0.1PG	3.12 ± 0.23 ^b^	0.01 ± 0.03 ^a^
0.8SG0.2PG	2.20 ± 0.42 ^c^	0.20 ± 0.15 ^a^
0.6SG0.4PG	0.67 ± 0.42 ^d^	2.23 ± 0.19 ^b^
0.4SG0.6PG	0.41 ± 0.28 ^d^	4.42 ± 0.31 ^c^
0.2SG0.8PG	0.25 ± 0.15 ^d^	7.03 ± 0.29 ^d^
PG	-	8.98 ± 0.73 ^e^

**Table 2:** Degree of substitution (DS) of GelMA samples obtained by the TNBS method and ^1^H-NMR. TNBS measurements were performed in triplicate. Different letters indicate statistically significant differences between groups (*p* < 0.05). Groups sharing at least one common letter are not significantly different (*p* ≥ 0.05), as determined by one-way ANOVA followed by Tukey’s multiple comparisons test.

GelMA Sample	GelMA DS (%)	
	TNBS	^1^H-NMR
SG	95.7 ± 2.1 ^a^	98.2
0.8SG0.2PG	96.5 ± 1.7 ^a^	95.2
0.6SG0.4PG	96.8 ± 1.2 ^a^	98.3
0.4SG0.6PG	96.0 ± 3.0 ^a^	95.0
0.2SG0.8PG	95.9 ± 1.0 ^a^	95.1
PG	96.8 ± 1.2 ^a^	97.8

**Table 3:** Melting enthalpy (∆H_m_) values, determined by the integration of the endotherms derived from salmon gelatin (SG endo) and porcine gelatin (PG endo) in GelMA samples (obtained using different SG-PG mixtures) at 10% *w*/*v* pH 7.4. (n = 3–9 per group). Different letters indicate statistically significant differences between groups (*p* < 0.05). Groups sharing at least one common letter are not significantly different (*p* ≥ 0.05), as determined by one-way ANOVA followed by Tukey’s multiple comparisons test.

GelMA Sample	ΔH_m_ (Jg^−1^)	
	SG Endo	PG Endo
SG	2.98 ± 0.32 ^a^	-
0.8SG0.2PG	1.42 ± 0.33 ^b^	ND
0.6SG0.4PG	0.76 ± 0.62 ^c^	0.72 ± 0.32 ^a^
0.4SG0.6PG	0.26 ± 0.11 ^c^	1.75 ± 0.63 ^b^
0.2SG0.8PG	0.26 ± 0.14 ^c^	4.12 ± 0.40 ^c^
PG	-	4.91 ± 0.89 ^c^

**Table 4:** Weibull kinetic parameters (α, β) obtained from release profile assays (R6G release from GelMA hydrogels). R^2^: coefficient of determination that determines fit accuracy.

GelMA Sample	Pre-Incubation Temperature (°C)	α (min)	β	R^2^
0.8SG0.2PG	37	52.3 ± 12.0	0.873 ± 0.03	0.998
	4	75.8 ± 14.0	0.849 ± 0.04	0.997
0.6SG0.4PG	37	60.3 ± 8.8	0.900 ± 0.08	0.998
	4	77.3 ± 6.3	0.860 ± 0.05	0.995
0.4SG0.6PG	37	47.2 ± 9.4	0.844 ± 0.06	0.997
	4	87.3 ± 9.8	0.791 ± 0.05	0.995
0.2SG0.8PG	37	58.3 ± 8.6	0.880 ± 0.03	0.998
	4	76.4 ± 8.7	0.871 ± 0.07	0.998

## Data Availability

Data will be available upon request.

## Supplementary Materials

The following supporting information can be downloaded at: https://www.mdpi.com/article/10.3390/gels12060540/s1, Figure S1. Representative oscillatory rheological curves of gelatin suspensions at 7% *w*/*v* pH 7.4 from SG-PG mixtures during cooling (3 °C/min). Arrows indicate Tgel values, determined by the G′-G″ crossover. Figure S2. Representative oscillatory rheological curves of GelMA suspensions at 7% *w*/*v* pH 7.4 from SG-PG mixtures during cooling (3 °C/min). Arrows indicate Tgel values, determined by the G′-G″ crossover. Table S1. Proximal analysis of gelatin samples. N.D: Not Detected. Table S2. Gelatin glycine, proline, and hydroxyproline contents of gelatin samples.
