# Supplementary material for: Designing Tunable GelMA Hydrogels by Integrating Mammalian and Non-Mammalian Gelatins
Source: Gels. 2026 Jun 15;12(6):540. doi: 10.3390/gels12060540 (PMC13298604; doi:10.3390/gels12060540)
Supplement: Supplementary file 1 [file gels-12-00540-s001.zip › gels-4352466-supplementary.pdf]

Supplemental information

Table S1. Proximal analysis of gelatin samples. N.D: Not Detected.

Sample	Fat (g/100g)	Protein (g/100g)	Ash (g/100g)	Non-N (g/100g)
PG	N.D	100.1 ± 0.0	0.3 ± 0.0	N.D
SG	N.D	94.7 ± 0.2	4.8 ± 0.2	4.8 ± 0.2

Table S2. Gelatin glycine, proline, and hydroxyproline contents of gelatin samples.

Sample	Glycine (g/100 g)	Proline (g/100 g)	Hydroxyproline (g/100 g)
PG	24.5 ± 0.6	12.0 ± 0.29	12.4 ± 0.2
SG	28.1 ± 1.5	10.7 ± 0.49	9.0 ± 0.6

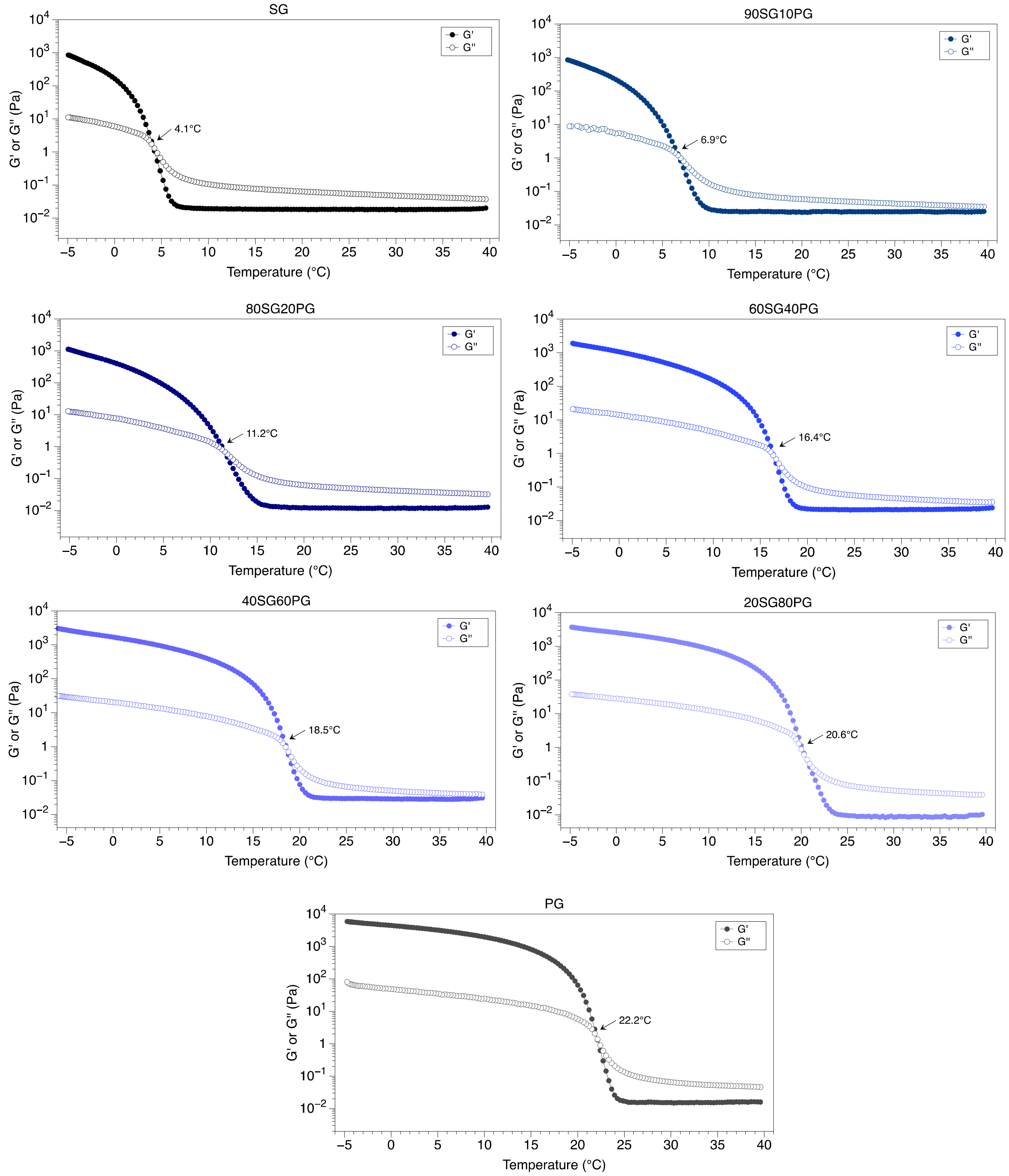

Figure S1. Representative oscillatory rheological curves of gelatin suspensions at 7% w/v pH 7.4 from SG-PG mixtures during cooling (3 °C/min). Arrows indicate Tgel values, determined by the G′-G″ crossover.

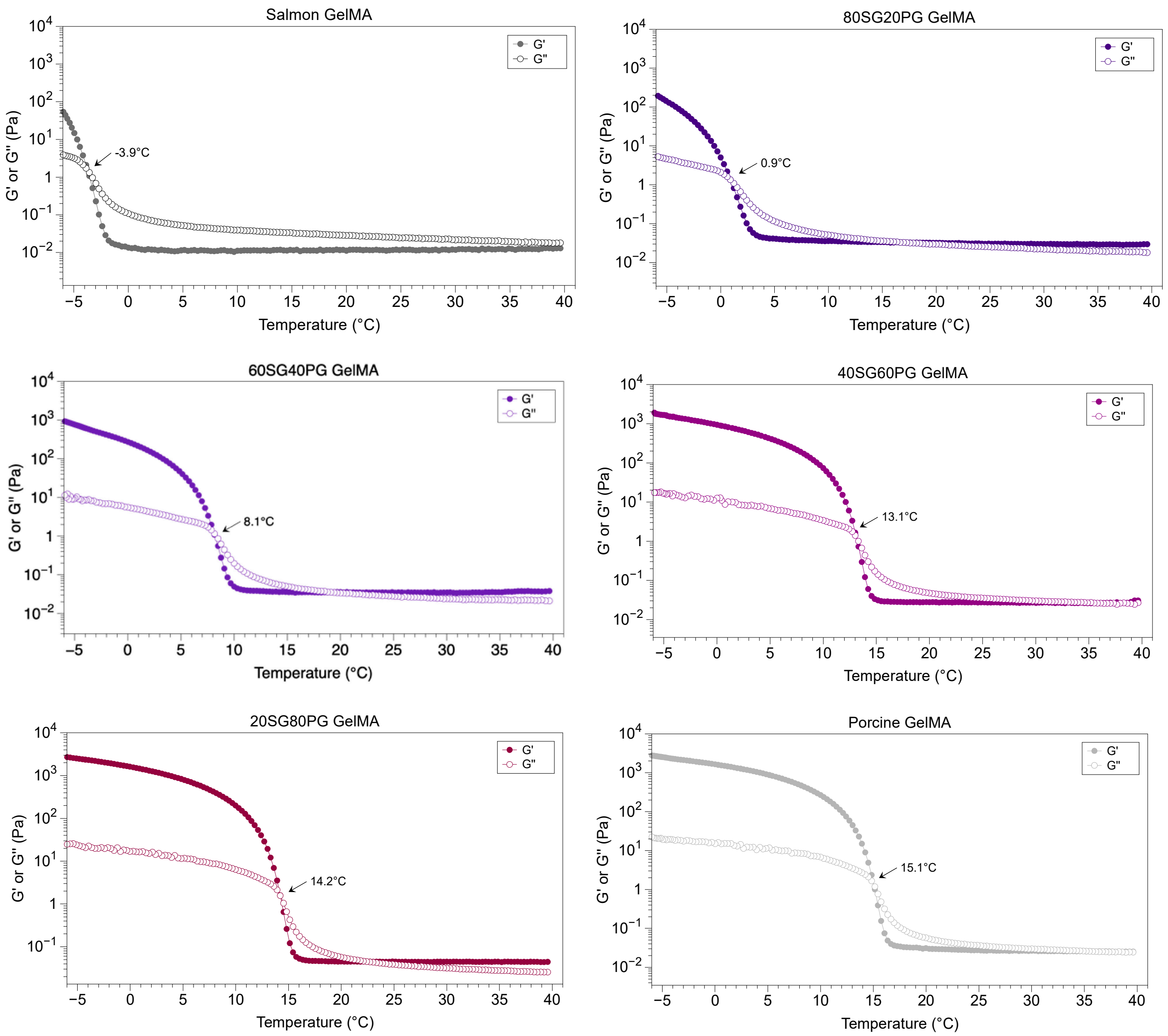

Figure S2. Representative oscillatory rheological curves of GelMA suspensions at 7% w/v pH 7.4 from SG-PG mixtures during cooling (3 °C/min). Arrows indicate Tgel values, determined by the G′-G″ crossover.
